# Silencing FYVE, RhoGEF, and PH domain containing 1 (FGD1) suppresses melanoma progression by inhibiting PI3K/AKT signaling pathway

**DOI:** 10.1080/21655979.2021.2005877

**Published:** 2021-12-07

**Authors:** Zehao Niu, Yan Li, Yujian Xu, Weiqian Jiang, Ran Tao, Youbai Chen, Yan Han

**Affiliations:** a Medical School of Chinese PLA, Beijing, China; b Department of Plastic and Reconstructive Surgery, The First Medical Center, Chinese PLA General Hospital, Beijing, China

**Keywords:** FGD1, PI3K, AKT, melanoma, prognostic factor, biomarker

## Abstract

Cutaneous melanoma is the leading cause of death among skin cancers despite the availability of diverse treatments. FGD1 plays an important role in multiple cancers, but how it works in cutaneous melanoma has not been illustrated. Thus, this study was intended to investigate the roles of FGD1 and its underlying mechanisms in cutaneous melanoma. Bioinformatics tools and quantitative real-time polymerase chain reaction (qRT-PCR) were used to analyze the expression of FGD1 in cutaneous melanoma. After the knockdown of FGD1 in melanoma cells, the proliferation, migration, and invasion of cells were analyzed by cell counting kit-8 (CCK8) assay, colony formation assays and transwell assays. Western blot was used to check the expression of key factors in PI3K/AKT pathway. In addition, nude mice models were used to study the role of FGD1 in melanoma development and metastasis in vivo. The data demonstrated that FGD1 was up-regulated and predicted a poor clinical outcome for cutaneous melanoma patients. Knockdown of FGD1 inhibited melanoma cell proliferation, migration, and invasion. The expressions of p-PI3K and p-AKT were significantly decreased, while the expressions of PI3K and AKT showed no marked difference in the knockdown group. Meanwhile, knockdown of FGD1 suppressed the development of melanoma in vivo. This study suggested that knockdown of FGD1 could block melanoma formation and proliferation by inhibiting PI3K/AKT signaling pathway. FGD1 might be a promising therapeutic target for melanoma.

## Introduction

Melanoma is derived from melanocytes, which synthesizes melanin [1]. The incidence of skin melanoma (SKCM) has been rising. It is the fifth most common cancer in adults and the deadliest form of skin cancer [2]. According to the latest data, the number of new cases in the United States was estimated at 100,350 and the estimated death toll was 6,850. Thanks to immunotherapy [3], BRAF/MAPK kinase (MEK) target therapy [4] and single-agent PD-1 (programmed cell death 1) blockade [5], 1-year survival rose from 42% in 2008 to 55% in 2013. Also, the overall survival rate increased by 7% annually from 2013 to 2017. Despite these advantages, these treatments have some limitations, such as drug resistance [2]. It is still necessary to continue to investigate other promising therapeutic targets.

Recently, bioinformatics tools have provided an insight into SKCM treatment [6,7]. For example, Su et al [8]. revealed that CXCL8 (C-X-C motif chemokine ligand 8), THBS1 (thrombospondin 1) and KIT (KIT proto-oncogene, receptor tyrosine kinase) might be the hub genes in metastasis process of SKCM. However, most of these studies were only based on bioinformatic analyses, making them less reliable owing to a lack of external and experimental validation. In the current study, bioinformatics analysis found that FGD1 could serve as a key gene in melanoma progression. It works as a guanine nucleotide exchange factor to control the activation of Rho GTPase, which is critical to normal development and tumor formation [9–11]. FGD1 positively regulates Cdc42 by exchanging GDP for GTP and evolves in cell morphology, gene transcription, cell cycle progression, cell adhesion, and so on [11–13]. Disfunction of FGD1 is known to be associated with a wide range of diseases. For example, the loss of function mutation of FGD1 leads to Aarskog-Scott syndrome [13]. Increased expression of FGD1 serves as an oncogene in hepatocellular carcinoma [14], breast cancer [15] and prostatic cancer. Furthermore, Wu et al. [16] demonstrated that FGD1 regulates osteosarcoma immune response by inhibiting phosphatase and tensin homologue (PTEN) gene activity. These studies indicated that FGD1 might play a key role in tumor formation. Similarly, the overexpression pattern of FGD1 was also found in cutaneous melanoma in our previous studies. However, the function of FGD1 and possible pathways have not been fully explored.

This work aimed to evaluate the role and underlying mechanisms of FGD1 in SKCM development. It was hypothesized that knockdown of FGD1 could refrain melanoma cell proliferation, migration, and invasion by inhibiting PI3K/AKT signaling pathway. To the best of our knowledge, this is the first study that comprehensively investigates the role of FGD1 in CM using both public datasets and experiments and proposed FGD1 as a potential therapeutic target for melanoma patients.

## 2. Materials and Methods

### 2.1. Bioinformatics analysis

The gene expression profile and clinical profile in the Cancer Genome Atlas (TCGA) and the Genotype-Tissue Expression (GTEx) were obtained from UCSC Xena browser (https://xenabrowser.net) [17]. Data form TCGA and GTEx were analyzed using the same packet in R software (version 4.0.3) to minimize the batch effects. GSE3189 and GSE15605 were obtained from the Gene Expression Omnibus (GEO) database (http://www.ncbi.nlm.nih.gov/geo/). GSE3189, based on platform GPL96, contained 7 normal skin samples, 18 nevi and 45 melanoma samples. In the present study, only melanoma and normal samples were analyzed. GSE15605, based on platform GPL570, contained 16 normal samples and 58 melanoma samples.

Selection of FGD1: This study began by identifying the differently expressed genes (DEgenes) in these databases by using ‘Limma’ package. The thresholds were *P*-value < 0.05 and log (fold change) > 2 for TCGA and GTEx, log (fold change) > 2 for GSE3189, and log (fold change) >1 for GSE15605. Secondly, data on survival and clinical characteristics were combined with the expression profile of TCGA database. The Kaplan–Meier analysis, the receiver operating characteristic (ROC) curve and cox analysis were performed in R software. Only genes with a prognostic value (*P* < 0.05 in Kaplan–Meier analysis, area under ROC curve > 0.6 and *P* < 0.05 in cox analysis) were selected for further analysis.

Pathway analysis: Based on the median expression values of FGD1, patients from TCGA database were divided into the high-expression group and low-expression group. DEgenes between the two groups were identified using ‘Limma’ package. Then, Kyoto Encyclopedia of Genes and Genomes (KEGG) pathway enrichment analysis was performed to further explore the underlying mechanism.

### 2.2. Patients and Samples

After being approved by the Ethics Committee of Chinese PLA General Hospital, 15 pairs of melanoma and normal tissues were collected. All patients were informed and signed informed consent forms.

### 2.3. Antibodies, reagents, and chemicals

The transfected plasmids were purchased from GeneChem (Shanghai, China). The FGD1 antibody was purchased from Novus Biologicals Littleton (working dilution 1:1000 for Western blot and 1:200 for IHC staining), the PI3K antibody from Cell Signaling Technology (working dilution 1:1000 for Western blot), the Phospho-PI3K antibody from Abcam (working dilution 1:500 for Western blot), the AKT antibody from Proteintech (working dilution 1:1000 for Western blot), the Phospho-AKT antibody from Proteintech (working dilution 1:2000 for Western blot) and the β-actin antibody from Proteintech (working dilution 1:10,000 for Western blot).

### 2.4. Histologic and Immunohistochemical Assessment

Melanoma and normal tissues were performed immunohistochemistry staining (IHC) as described in related studies [18]. Shortly, samples were fixed in 10% buffered formalin and embedded in paraffin. After deparaffinization, rehydration and antigen retrieval, tissues were incubated with 1:200 diluted anti-FGD1 antibody at 4°C overnight. After being washed with phosphate-buffered saline (PBS) for 3 times, tissues had been incubated with a secondary antibody (biotinylated goat anti-mouse secondary antibody, dilution 1:200, abnova) for 30 minutes. Then, tissues had been cultivated with 3,3‐Diaminobenzidine (DAB) for 1 minute and counterstained with hematoxylin. For hematoxylin and eosin (HE) staining [19], lung tissues were fixed in 10% buffered formalin and embedded in paraffin. Sections at 5 µm thickness were stained with hematoxylin and eosin solution followed by dehydration with graded alcohol and clearing in xylene.

### 2.5. Cell culture and transfection

Human melanoma cell line: A375 was purchased from American Type Culture Collection (ATCC, America); M14 cell line was purchased from Shanghai GuanDao Biological Engineering Company (Shanghai, China) with STR certification; SK-MEL-1 cell line was purchased from National Infrastructure of Cell Line Resource. Human immortalized keratinocytes cell line (Hacat) was purchased from Shanghai Guandao Biological Engineering Company with STR certification. All cells were cultured in Roswell Park Memorial Institute-1640 (RPMI-1640) +10% fetal bovine serum and maintained at 37°C in a 5% CO_2_ atmosphere. A375 and M14 cell lines were assigned as follows: KD-con group (cells transfected with blank plasmid used in FGD1 knockdown), KD1 group (cells transfected with shRNA1), KD2 group (cells transfected with shRNA2). Transfection was performed according to the manufacturer's guidelines [20]. Briefly, the transfection procedure was performed with the best multiplicity of infection value (MOI = 10). The media was changed 16 hours after transfection and the efficacy of transfection was observed under a fluorescence microscope.

### 2.6. RNA isolation and validation

The real-time quantitative PCR (qRT-PCR) was performed to determine relative expression levels of the genes. Total RNA was extracted with TRIzol reagent 24 hours after transfection. The concentration of RNA was determined by ultraviolet spectrophotometry. Then, RNAs were reverse transcribed into cDNAs (50 ng/µl) using commercial cDNA reverse transcription kit. Finally, SYBR (Takara, Japan) was used to evaluate the mRNA expression levels [21]. GAPDH (glyceraldehyde-3-phosphate dehydrogenase) was used as the internal reference. Primer sequences were listed as follows: GAPDH: forward, GGA AGC TTG TCA TCA ATG GAA ATC; reverse, TGA TGA CCC TTT TGG CTC CC; FGD1: forward, AAA ATG AAC CCT TGG TGC TG; reverse, GGC TGA AGT ACC AGC TGA GG; Bcl-2 (BCL2 apoptosis regulator): forward, GGT GAA CTG GGG GAG GAT TGT; reverse, CCA GGA GAA ATC AAA CAG AGG CBC; Bax (BCL2 associated X): forward, CGG GTT GTC GCC CTT TTC TA; reverse, GAG GAA GTC CAA TGT CCA GCC. All primers were synthesized by Servicebio Technology Company (Wuhan, China). The PCR program [22] was set as: 10 min at 95°C, followed by 45 cycles of 95°C for 10 s, 60°C for 30 s and 72°C for 20 s. The expression of target genes was calculated using 2^−ΔΔCt^ method.

### 2.7. Western blot

For Western blotting, the cells were lysed and total protein was extracted by RIPA lysis buffer supplemented with phosphatase and protease inhibitor. The concentration of protein was determined by ultraviolet spectrophotometry. Then, equal amounts (20 μg) of protein were separated by sodium dodecyl sulfate polyacrylamide gel electrophoresis. Proteins were then transferred to polyvinylidene difluoride membranes and blocked by 5% skim milk for 1 hour. The membranes were allowed to react with the primary antibodies at 4°C. After being rinsed by Tween-20 (TBST) 3 times, the membranes reacted with secondary antibodies for 1 hour at room temperature. Finally, the Western blotting bands were visualized using the Amersham Imager AI680 chemiluminescence system [23].

### 2.8. Cell proliferation assessment

Cell proliferation was measured using cell counting kit-8 (CCK-8) proliferation assay. Approximately 2,000 cells were seeded into each well of a 96-well plate. At the appointed time point (24 h, 48 h, 72 h and 96 hours after inoculation), 10 μl of CCK-8 solution (Solarbio, China) was added into each well. After incubation at 37°C for 3 hours, the optical density value of excitation light was detected with a microplate reader [24].

### 2.9. Cell migration and invasion assessment

Transwell chambers with 8.0 μm pores were used to determine the migration and invasion of melanoma cells. For migration assay, transfected cells were washed with PBS twice and seeded in the upper chamber. FBS-free medium was added into the upper chamber and the RPMI-1640 culture medium containing 10% FBS medium was added into the lower chamber. After being cultured for 24 hours, cells of the upper chamber were removed using a swap. Migrated cells had been fixed by 4% paraformaldehyde for 10 minutes and stained with crystal violet. Five visual fields were randomly selected and the number of cells were calculated under an optimal microscope at 200× magnification. For invasion assay, the upper compartment of the transwell chamber was precoated with Matrigel [24].

### 2.10. Colony formation assay

Transfected cells were seeded in 6-well plates (1000 cells/well). After being cultured for 14 days, culture medium was discarded and cells were washed with PBS. Then, cells had been fixed with methanol for 15 minutes and stained with crystal violet. Clones with more than 50 cells were counted [24].

### 2.11. In vivo studies

All the animal experiments were approved by the Ethics Committee of Chinese PLA General Hospital. For tumor xenograft model [25], nude mice (BALB/c, aged 6 weeks) received subdermal injection of 5 × 10^6^ transfected A-375 cells. All mice were divided into two groups (n = 6 for each group). The tumor length (L) and tumor width (W) were measured, and the tumor volume was calculated by the following formula: L*(W)^2^/2. Tumor growth was measured every week. Tumor weight was measured at the end of the experiment. For metastasis model [26], 2 × 10^6^ transfected A375 cells were injected via tail vein injection of nude mice. After 21 days, animals were sacrificed and the lungs were removed. Lung tissues were sliced and stained with HE as aforementioned and the number of metastases were calculated.

### 2.12. Statistical analysis

Data in this study were shown as the mean ± standard deviation. Statistical analysis was performed in SPSS software. Student’s t-test was used to compare the difference between the two groups and *P* < 0.05 was considered statistically significant.

## 3. Results:

FGD1 is a guanine nucleotide exchange factor to control the activation of Rho GTPase, which is critical to normal development and tumor formation. In this study, FGD1 was found to be significantly upregulated in melanoma tissue and the expression of FGD1 was related to the survival of melanoma patients. Furthermore, FGD1 was identified as an independent prognosis predictor in univariate and multivariate analysis. Thus, it was assumed that FGD1 might play an important role in melanoma formation and progression. To investigate the underlying mechanism, shRNA was used to knockdown the expression of FGD1 in two melanoma cell lines. The results showed that knockdown of FGD1 significantly decreased proliferation, migration, invasion and colony formation of melanoma cells. Knockdown of FGD1 also influenced the expression of apoptosis-related genes. In the nude mice model, knockdown of FGD1 inhibited the growth of tumor tissue. Knockdown of FGD1 inhibited the activation of PI3K/AKT pathway. These results demonstrated that FGD1 might serve as a novel target for the treatment of cutaneous melanoma.

### 3.1. FGD1 is up-expressed in SKCM patients and related to poor prognosis.

**Figure 1.**
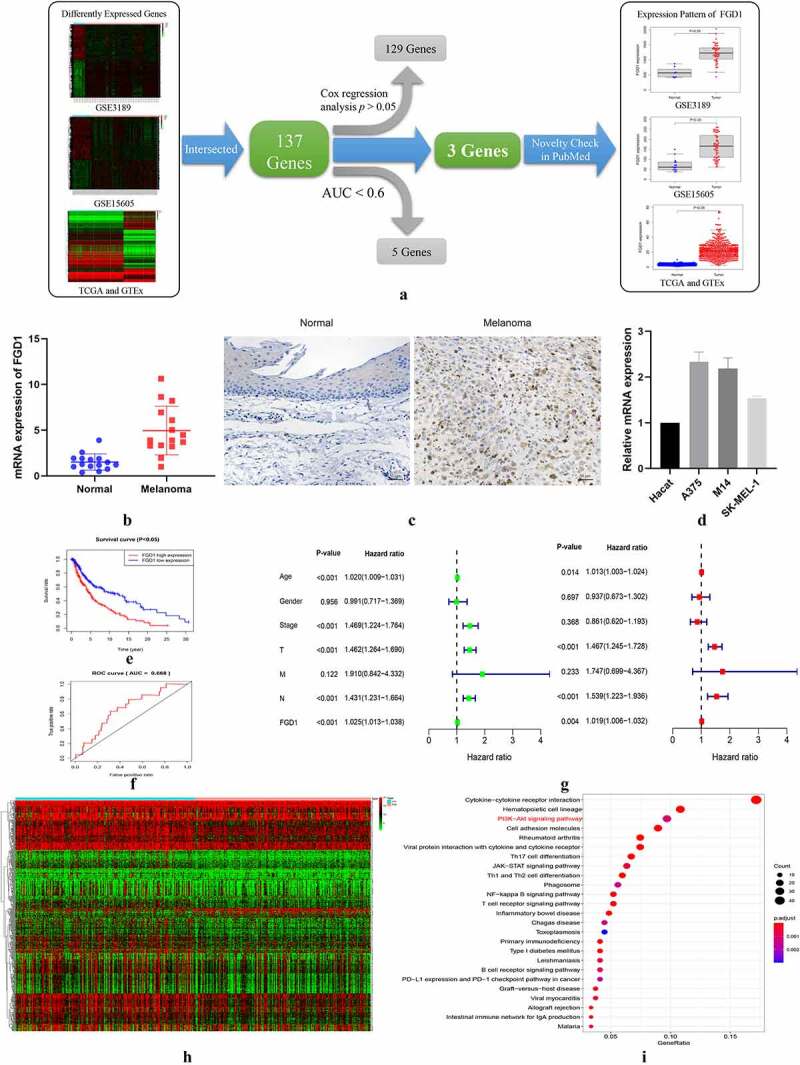
A. Flowchart for FGD1 (FYVE, RhoGEF and PH domain containing 1) selection. Differently expressed genes from GSE3189, GSE15605 and TCGA (The Cancer Genome Atlas) and GTEx (the Genotype-Tissue Expression) datasets were identified, then, 137 intersected genes were included for further study. After Kaplan–Meier analysis, the receiver operating characteristic (ROC) curve, Cox analysis and novelty check in PubMed, FGD1 was selected as target gene; B. The mRNA expression levels of FGD1 in melanoma and normal tissues were detected by real-time quantitative PCR; C. Representative IHC (immunohistochemistry) staining of FGD1 in normal and melanoma tissues (200 × magnification); D. Relative mRNA expression level of FGD1 in Hacat (human immortalized keratinocytes cell line) and 3 melanoma cell lines; E. Correlation between FGD1 expression and patient’s survival; F. ROC analysis of FGD1; G. Forest plot of univariate and multivariate Cox analysis; H. Heatmap of differently expressed genes between FGD1 high-expressed group and low-expressed group. I. Kyoto Encyclopedia of Genes and Genomes (KEGG) pathway enrichment analysis of differently expressed genes

Based on the preset thresholds, 1844 DEgenes were identified in TCGA and GTEx database (684 up-regulated genes and 1160 down-regulated genes), 3274 DEgenes in GSE3189 database (1687 up-regulated genes and 1587 down-regulated genes), and 2604 DE genes in GSE15605 database (1050 up-regulated genes and 1554 down-regulated genes). A total of 137 intersected genes were included in the following study. After Kaplan–Meier analysis, the receiver operating characteristic (ROC) curve and cox analysis, only 3 genes were qualified as prognosis-related genes, namely FGD1, TUBB4A (tubulin beta 4A class IVa) and DLL3 (delta-like canonical Notch ligand 3) (Figure 1(a)). After novelty check in PubMed database, FGD1 was selected as the target gene.

The distribution of FGD1 in normal tissues was shown in Figure S1, As shown in distribution map and box plot, FDG1 was expressed in almost all the tissue and was lowly expressed in normal tissues and organs. As shown in Figure 1(a), FGD1 was highly expressed in cutaneous melanoma compared with that in normal skin tissues. Compared with similar studies [23,24], the expression level of FGD1 was evaluated in 3 independent research groups, which shows the conclusion is believable. The expression level of FGD1 was further validated in clinical samples. Figure 1(b) shows that the mRNA expressions level of FGD1 were up-regulated in melanoma tissues. As revealed by immunohistochemical staining (Figure 1(c)), FGD1 staining intensity was markedly higher in melanoma tissues. Figure 1(d) shows the expression of FGD1 was significantly up-regulated in 3 melanoma cell lines (A375, M14 and SK-MEL-1) compared with that in HaCat cell line. In Kaplan–Meier survival analysis, higher expression of FGD1 was related to poorer prognosis (Figure 1(e)). The area under ROC curve was 0.668, indicating the prognosis model based on the expression of FGD1 was fair (figure 1(f)). Furthermore, 346 patients with full clinical characteristics, including age, gender, stage, T, M, N stage were included in cox analysis. Univariate analysis showed that age, stage, T, N stage and FGD1 were associated with prognosis (Figure 1(g)). Multivariate cox analysis (Figure 1(g)) showed that FGD1 was an independent predictor of prognosis along with age, T stage and N stage. Based on the median value of FGD1, 598 DE genes were identified between high-expression and low-expression groups, including 48 up-regulated genes and 550 down-regulated genes (Figure 1(h)). In KEGG analysis, these genes were mainly enriched in cytokinecytokine receptor interaction (44/262), hematopoietic cell lineage (26/262) and PI3K-AKT singling pathway (25/262) (Figure 1(i)).

### 3.2. Knockdown of FGD1 in A375 and M14 cell lines.

**Figure 2.**
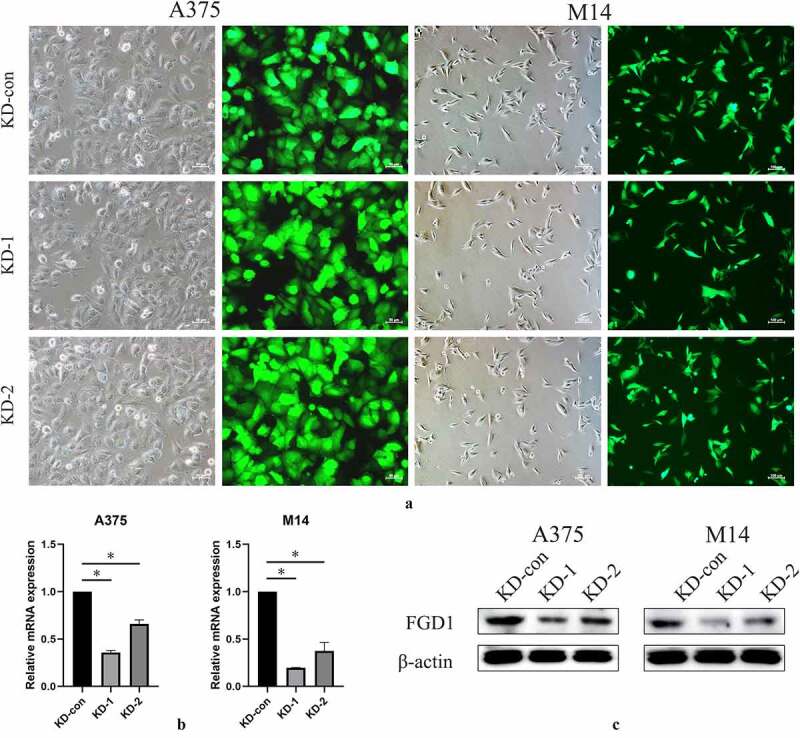
A. shRNA plasma was successfully transfected into cells and green fluorescence can be seen in almost all cells. Magnification is 200× for A375 cells and 200× for M14 cells; B. Relative FGD1 (FYVE, RhoGEF and PH domain containing 1) mRNA expression level was evaluated by qRT-PCR assay; C. Protein level of FGD1 (FYVE, RhoGEF and PH domain containing 1) was evaluated by Western blot. * means *P < *0.05

To investigate the underlying mechanism in SKCM cell lines, two FGD1 shRNAs were constructed and the relative mRNA expression was tested by qRT-PCR and Western blot. Fluorescence showed that the shRNA plasmid was successfully transfected into A375 and M14 cells (Figures 2(a), 200× for A375 and 100× for M14 cells). As a consequence, the expression of FGD1 was significantly downregulated. The shRNA1 group achieved higher knockdown efficacy and was chosen for subsequent experiments (Figures 2(b,c)).

### 3.3. Knockdown of FGD1 inhibited melanoma cell migration, invasion, proliferation and colony formation, and promoted the expression of apoptosis-related genes.

**Figure 3.**
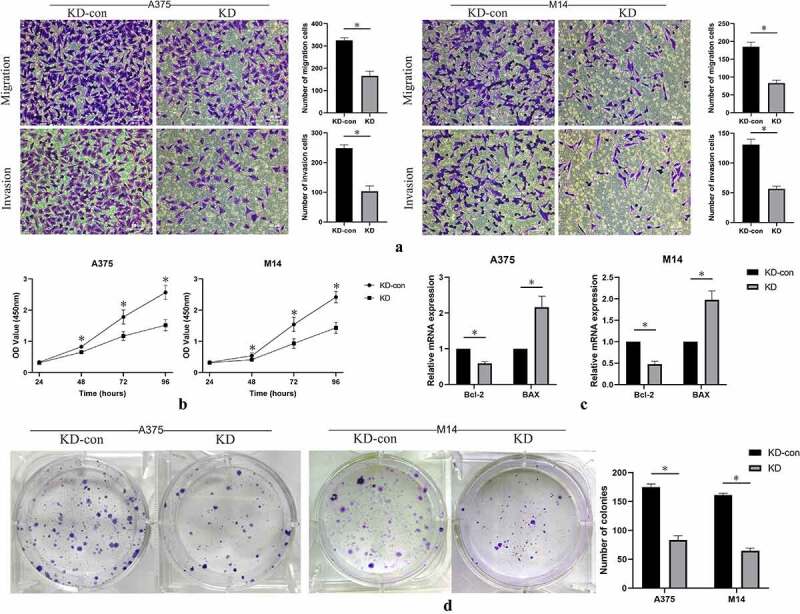
A. Cell migration and invasion were performed in A375 and M14 cells after transfection. The number of migrated or invaded cells was counted by analyzing photographs at 200 × magnification in five random fields per chamber; B. cell counting kit-8 (CCK-8) analysis of knockdown group and control group in 24, 48, 72, 96 hours; C. Relative mRNA expression of apoptosis-related genes, including Bax (BCL2 associated X) and Bcl-2 (BCL2 apoptosis regulator) were detected by qRT-PCR. D. Cell growth was detected by colony formation assay in transfected cells. * means *P < *0.05

Cell migration and invasion were detected using transwell assay. Compared with the control group, inhibited expression of FGD1 significantly decreased the number of migration and invasion cells (Figure 3(a)). These data suggest that FGD1 was closely related to the migration and invasion of melanoma cells.

Cell proliferation was measured by CCK-8 assay. The results showed that the optical density value in FGD1 knockdown group was significantly lower than that in the control group (Figure 3(b)). The difference gradually appeared in approximately 48 hours after incubation. This result indicated that knockdown of FGD1 significantly decreased the proliferation ability of melanoma cells.

Colony-formation ability of A375 and M14 cells after knockdown of FGD1 were evaluated. As shown in Figure 3(d), knockdown of FGD1 significantly decreased the number of colonies in both A375 and M14 cells (P < 0.05). These results indicated that FGD1 was associated with the ability of colony of melanoma cells.

In previous studies, FGD1 was reported to be related to cell apoptosis [16]. Thus, the expressions of apoptosis-related genes were evaluated by qRT-PCR assay. Knockdown of FGD1 significantly decreased the expression of Bcl-2, but significantly increased the expression of BAX (Figure 3(c)).

### 3.4. Knockdown of FGD1 inhibited PI3K/AKT signaling pathway.

**Figure 4.**
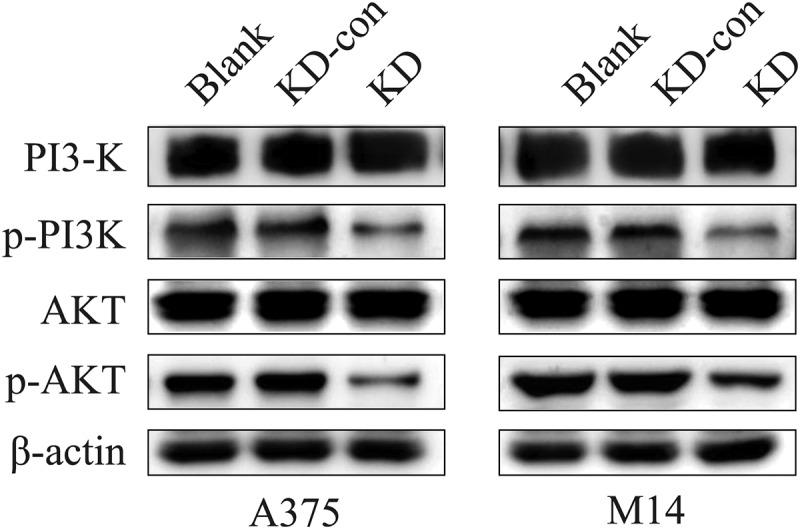
Knockdown FGD1 (FYVE, RhoGEF and PH domain containing 1) might regulated the physiological state of cells via PI3K/AKT signaling pathway. The protein level of key factors in this pathway was detected by Western blot

As indicated in KEGG analysis and previous reports, PI3K/AKT signaling pathway is closely related to melanoma [27]. Thus, this study further investigated whether the expression of FGD1 was related to PI3K/AKT signaling pathway activation. Western-blot was performed to access the expression of total PI3K (PI3K), Phospho-PI3K (p-PI3K), total AKT (AKT) and Phospho-AKT (p-AKT), which were key factors in this pathway. As shown in Figure 4, the expressions of p-PI3K and p-AKT were significantly decreased in the FGD1-KD group compared with blank and KD-con group. However, the expressions of PI3K and AKT hardly changed. These results indicated that knockdown of FGD1 inhibited the activation of PI3K/AKT signaling pathway.

### 3.5. Knockdown of FGD1 inhibited melanoma growth and lung metastasis in vivo

**Figure 5.**
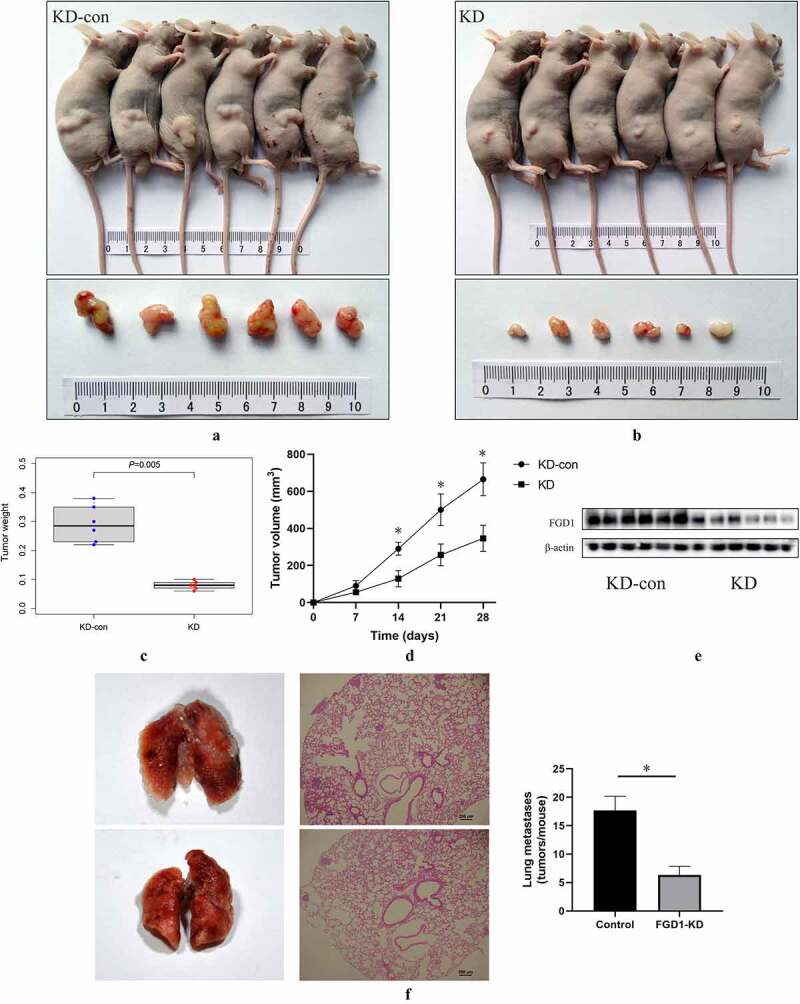
Knockdown FGD1 (FYVE, RhoGEF and PH domain containing 1) inhibited tumor growth in vivo. A, B. Tumors in control group and FGD1 knockdown group were isolated and pictured 4 weeks after injection; C. Tumor weight was calculated in control group and FGD1 knockdown group; D. Tumor volume was calculated in control group and FGD1 knockdown group. E. Protein level of FGD1 was evaluated by western bolt; F. FGD1 knockdown significantly reduced tumor metastasis in A375 melanoma lung metastasis model in vivo. * means *P < *0.05

A subcutaneous xenograft model was constructed to identify the role of FGD1 in melanoma. The results showed that the downregulated expression of FGD1 significantly inhibited the growth of melanoma in vivo (Figure 5(a)). Four weeks after injection, the tumor weight (Figure 5(b)) of control group was 0.292 ± 0.064 g, significantly higher than that in the knockdown group (0.080 ± 0.014 g). Moreover, the tumor size of control group was 664.71 ± 88.94 mm^3^, significantly larger than that in the knockdown group (346.25 ± 70.61 mm^3^). Compared with the control group, the protein level of FGD1 tumor was significantly lower in FGD1-KD group (Figure 5(e)). Next, a metastasis model was employed to assess FGD1-knockdown of lung tumorigenesis after tail vein injection of melanoma cells. As shown in figure 5(f), compared with the control group, knockdown of FGD1 decreased the number of lung metastasis [17.67 ± 2.52 versus 6.33 ± 1.53 (tumors per lung; p < 0.05)].

## Discussion

FGD1 is located on Xp11.21 and consists of 18 exons. It has a catalytic region that is composed of Dbl homology (DH) domain and pleckstrin homology 1 (PH1) domain [12]. It is well documented that FGD1 can work as a guanine exchange factor and is responsible for activating Cdc42 (cell division cycle 42) followed by activating a number of oncogene pathways [13,28]. It is reported that oncogene property of FGD1 have been found in several tumors. For example, Whitehead et al [29]. found that FGD1 was able to cause tumorigenic transformation of NIH 3T3 fibroblasts by activating Cdc42 and other signaling pathways. Wu et al [16]. found that FGD1 promoted osteosarcoma progression and regulated tumor immune response. Zeng et al [14]. noted that FGD1 regulated cell morphology, autophagy and mitochondrial function in hepatocellular carcinoma. In a bio-informatic study, Cai et al [30]. also found FGD1 played an important role in HCV (Hepatitis C) infection and alcohol consumption, and might be a potential therapy target for hepatocellular carcinoma.

In this study, with the use of bioinformatic tools, q-PCR and IHC, FGD1 was found to be highly expressed in melanoma tissues. Thus, the expression level of FGD1 was knock downed by shRNA in two melanoma cell lines. Compared with the control group, knockdown of FGD1 inhibited melanoma cell migration and invasion. These results can be interpreted in part due to the reduction of invadopodias, which were distributed on the surface of tumor cells. Invadopodia is a kind of specialized F-actin-based protrusions that are able to induce extracellular matrix degradation and enable cell migration and invasion [31–34]. The formation of invadopodia is associated with patients’ prognosis in invasive tumors, such as head and neck squamous cell carcinoma [35] and bladder cancer^28^. Further, it has been proven that FGD1 is associated with invadopodia formation in prostate cancer cell line (P3, Ayala et al [31]), breast cancer cell line (MDA-MB-231, Ayala et al [31]) and melanoma cell line (A375, Ayala et al [31]). Targeting invadopodia formation is an effective way to block cancer progression. For example, Stoletov et al [36]. noted targeting the key signaling hub in invadopodia (Scr) prevented cancer cell from escaping the lung vasculature and distant metastases. Similar therapeutic potential was noted in breast cancer [37] and pancreatic cancer [38].

With the use of bioinformatic tools, we also found differently expressed genes between FGD1 high-expression and low-expression groups were mainly enriched in PI3K/AKT signaling pathway. The results of Western blot showed that the expression of p-PI3K and p-AKT significantly decreased after knockdown of FGD1. These findings were similarly to previous studies. For example, Wu et al [16]. reported that FGD1 functions as an oncogene by inhibiting PTEN activity and activating PI3K/AKT pathway in osteosarcoma. After knocking down FGD1 in U-2OS, MG63 and MNNG/HOS cells, the content of p-AKT and pFOXO1 significantly decreased.

During these years, the important role of PI3K/AKT pathway in tumor formation and progression has been well illustrated. It can be activated by multiple factors, including growth factors and cytokines. After phosphorylation-mediated activation of PI3K and AKT, several key downstream effectors were also activated, including mTOR complex 1 (mTORC1), glycogen synthase kinase 3 (GSK3), and forkhead box protein O1 (FoxO1). PI3K/AKT pathway is associated with cell proliferation, invasion, apoptosis and autophagy [39–41]. Thus, in this study, the influence of FGD1 on cell apoptosis was further evaluated. The results showed that knockdown of FGD1 significantly decreased the expression of Bcl-2 and increased the expression of BAX, indicating the apoptosis pathway was activated. These results are consistent with Long et al [42], Dang et al [43] and Bai et al [44]. The influence of FGD1 on cell proliferation can also be explained by the inhabitation of PI3K/AKT pathway [45].

As well illustrated in previous literature, PI3K/AKT pathway has extensive connections with melanoma. Yang et al [46]. reported that downregulation of lncRNA MIAT inhibited melanoma migration and invasion through PI3K/AKT pathway. Yang et al [47]. reported that silencing MCM7 promoted cell autophagy and apoptosis by inactivating AKT1/mTOR signaling pathway. Thus, inhibitors targeting PI3K/AKT pathway have become a hotspot in tumor therapy [48]. These compounds include Rapamycin [49] (PI3k, AKT and mTOR inhibitor), Itraconazole [50] (PI3k and mTOR inhibitor) and PI-103(PI3K and mTOR inhibitor [51]). Considering the connections between FGD1 and PI3K/AKT pathway, it is reasonable to believe that FGD1 would show important clinical significance.

## Limitations

There are several limitations to the study. Firstly, the expression level of FGD1 was validated in 15 pairs of tissues and 4 cell lines. The validations in large-scale samples are still in need. Secondly, although oncogene properties of FGD1 are associated with PI3K/AKT pathway, other mechanisms still need to be explored.

## Conclusion:

In conclusion, FGD1 was found to be over-expressed in melanoma and could be considered as an independent prognosis factor for SKCM patients. Knockdown of FGD1 could inhibit melanoma cell proliferation, colony formation, migration, and invasion. Such oncogene properties are associated with the activation of PI3K/AKT pathway. Collectively, FGD1 may serve as a potential therapeutic target in melanoma and may provide more information for personalized medicine.

## Supplementary Material

Supplemental Material

## Highlights

(1) FGD1 was highly expressed in cutaneous melanoma tissues than normal tissues.

(2) Silencing FGD1 inhibited the migration, invasion, colony formation, and proliferation ability of melanoma cells.

(3) Oncogene property of FGD1 is closely related to the activation of PI3K/AKT pathway.

(4) FGD1 may serve as a novel therapeutic target in cutaneous melanoma.
