# Supplementary figures and images for: Silencing FYVE, RhoGEF, and PH domain containing 1 (FGD1) suppresses melanoma progression by inhibiting PI3K/AKT signaling pathway
Source: Bioengineered. 2021 Dec 7;12(2):12193–205. doi: 10.1080/21655979.2021.2005877 (PMC8810171; doi:10.1080/21655979.2021.2005877)

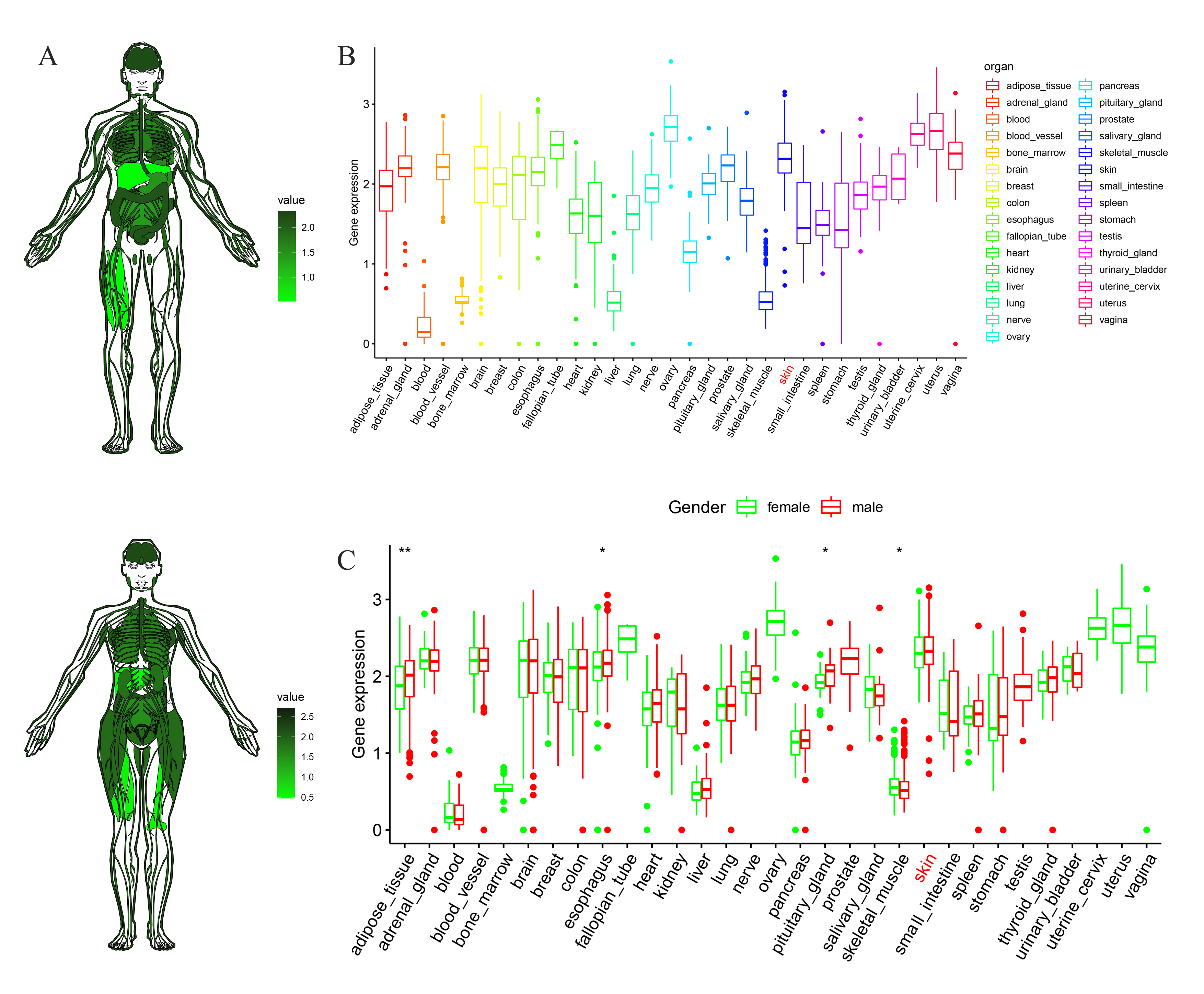

Supplement: Supplemental Material [file KBIE_A_2005877_SM0556.tif]
